# NT-ProBNP Levels in Saliva and Its Clinical Relevance to Heart Failure

**DOI:** 10.1371/journal.pone.0048452

**Published:** 2012-10-31

**Authors:** Jared Yong Yang Foo, Yunxia Wan, Karam Kostner, Alicia Arivalagan, John Atherton, Justin Cooper-White, Goce Dimeski, Chamindie Punyadeera

**Affiliations:** 1 The Australian Institute for Bioengineering and Nanotechnology, University of Queensland, Brisbane, Queensland, Australia; 2 School of Medicine, University of Queensland, Brisbane, Queensland, Australia; 3 Department of Cardiology, Mater Adult Hospital, Brisbane, Queensland, Australia; 4 Department of Cardiology, Royal Brisbane and Women’s Hospital, Brisbane, Queensland, Australia; 5 School of Chemical Engineering, University of Queensland, Brisbane, Queensland, Australia; 6 Chemical Pathology, Princess Alexandra Hospital, Brisbane, Queensland, Australia; The University of Texas Health Science Center, United States of America

## Abstract

**Background:**

Current blood based diagnostic assays to detect heart failure (HF) have large intra-individual and inter-individual variations which have made it difficult to determine whether the changes in the analyte levels reflect an actual change in disease activity. Human saliva mirrors the body’s health and well being and ∼20% of proteins that are present in blood are also found in saliva. Saliva has numerous advantages over blood as a diagnostic fluid which allows for a non-invasive, simple, and safe sample collection. The aim of our study was to develop an immunoassay to detect NT-proBNP in saliva and to determine if there is a correlation with blood levels.

**Methods:**

Saliva samples were collected from healthy volunteers (n = 40) who had no underlying heart conditions and HF patients (n = 45) at rest. Samples were stored at −80°C until analysis. A customised homogeneous sandwich AlphaLISA^(R)^ immunoassay was used to quantify NT-proBNP levels in saliva.

**Results:**

Our NT-proBNP immunoassay was validated against a commercial Roche assay on plasma samples collected from HF patients (n = 37) and the correlation was r^2^ = 0.78 (p<0.01, y = 1.705× +1910.8). The median salivary NT-proBNP levels in the healthy and HF participants were <16 pg/mL and 76.8 pg/mL, respectively. The salivary NT-proBNP immunoassay showed a clinical sensitivity of 82.2% and specificity of 100%, positive predictive value of 100% and negative predictive value of 83.3%, with an overall diagnostic accuracy of 90.6%.

**Conclusion:**

We have firstly demonstrated that NT-proBNP can be detected in saliva and that the levels were higher in heart failure patients compared with healthy control subjects. Further studies will be needed to demonstrate the clinical relevance of salivary NT-proBNP in unselected, previously undiagnosed populations.

## Introduction

Heart failure (HF) is a global health problem, associated with poor clinical outcomes and substantial economic burden to the healthcare system [1], [2]. Approximately, 23 million people worldwide are living with HF [2]. The population estimates of HF prevalence ranges between 2 and 10%, with a higher prevalence in the elderly [3].

Plasma/serum concentrations of natriuretic peptides, N-terminal proB-type natriuretic peptide (NT-proBNP, 76 AA) or B-type natriuretic peptide (BNP, 32 AA) are currently used to diagnose HF [4]–[7]. Several companies including Roche Diagnostics commercialise NT-proBNP immunoassays targeting various fragments of the NT-proBNP molecule (middle part of the NT-proBNP molecule is glycosylated). Therefore, the NT-proBNP results are not comparable across laboratories [8]–[10]. Current blood-based ‘sandwich’ immunoassays use monoclonal and polyclonal antibodies targeting different epitopes to quantify plasma levels of NT-proBNP and BNP [11]–[14]. This may complicate interpretation of plasma levels of NT-proBNP/BNP for diagnosing and monitoring HF, especially if a patient accesses different laboratory services that use different assays/platforms. These differences will only be minimised with improved understanding of the molecular forms and glycosylation patterns of NT-proBNP and BNP in the circulation.

Human saliva composition reflects our body’s health and well being and about 20% of proteins that are present in the blood are also found in saliva [15], which highlights the diagnostic potential of saliva. Saliva does not clot like blood, and its collection is non-invasive [16]–[18]. Saliva samples are relatively easy to handle in comparison to blood collection and processing thereby decreasing the risk of contracting blood-borne infectious organisms [19]–[21]. Furthermore, avoiding the need for a phlebotomist enables multiple saliva sample collections within a day by unskilled people.

The half-life of BNP is approximately 20 minutes and that of NT-proBNP is around 60–90 minutes [22], [23]. Hence, NT-proBNP clearance from blood is slower than its counterpart BNP, allowing possible movement of the former molecule into the saliva through various routes, but mainly via the gingival crevicular fluid [24]. We hypothesise that the relatively long half-life of NT-proBNP in circulation enables substantial movement of NT-proBNP from blood into saliva. The aims of our study were to develop an immunoassay to detect NT-proBNP in saliva and to determine if there is a correlation with plasma levels.

**Table 1:** Characteristics of HF patients and healthy controls.

Parameter	HF patients (n = 45)	Healthy controls (n = 40)	Statistics
Age	73 (53–88)	56 (40–71)	P_M_<0.0001*
Gender (M: F)	23∶22	20∶20	P_C_ = 0.919
Body Mass index (kg/m^2^)	29.14 (20–42.6)	25 (20–37)	P_M_ = 0.0194*
NYHA Classification	3	0	P_M_<0.0001*
Systolic blood pressure (mm Hg)	125 (93–158)	n/a	n/a
Diastolic blood pressure (mm Hg)	71 (45–86)	n/a	n/a
Heart rate (bpm)	71 (46–80)	n/a	n/a

Results are shown as median (min-max) for the data without normal distribution.

P_M_ = p-value for 2-tailed Mann-Whitney *U* test.

P_C_ = p-value for 2-sided Chi-square test.

* Significant at 0.05 level.

n/a = Not applicable.

## Materials and Methods

### 2.1 Participants

This research was approved by the University of Queensland Medical Ethical Institutional Board and the Mater Hospital Medical Ethical Review Board. All participants were >18 years of age and gave written informed consent before donating samples for our study. We recruited two cohorts of volunteers: patients with symptoms and/or signs of HF at varying clinical stages (with left ventricular ejection fraction <40%) from a general cardiology department and healthy control subjects. HF diagnosis was confirmed by the cardiologist from Mater Adult Hospital according to the Guidelines for the prevention, detection and management of chronic HF in Australia [5]. Patients with concomitant disease states that might alter salivary NT-proBNP concentrations (e.g. rheumatoid arthritis, inflammatory bowel disease, Sjogren’s syndrome, Raynaud’s disease) were excluded. All subjects were asked to refrain from exercise for 24 hours prior to sample collection. Exclusion criteria included the existence of any comorbid oral disease (e.g periodontal disease and gingivitis), autoimmune, infectious, musculoskeletal disease, malignancy, and recent operation or trauma. The subjects were of European, African and Asian descent, and had no symptoms of fever and/or respiratory tract infection. The salivary NT-proBNP and plasma concentrations from the HF patients were then compared with the clinical diagnostic criteria used by cardiologists in order to determine the diagnostic utility of the salivary NT-proBNP immunoassay.

**Table 2:** Performance characteristics of our NT-proBNP immunoassay.

Analyte	% Recovery	% Intra-assay variation(± Std error)	% Inter-assay variation(± Std error)	Limit of detection (LOD)
NT-proBNP	85%	7.17 (0.75)	4.46 (0.59)	16 pg/mL

### 2.2 Samples

Blood samples were collected into EDTA tubes (Greiner VACUETTE® # 454023, Greiner Bio-one, Graz, Austria) and then immediately centrifuged at 3000×g at RT for 10 minutes. The plasma samples were divided into aliquots, and stored at −80°C until analysed. Saliva samples were collected in sterile urine containers (Sarstedt, Australia) and stored at −80°C until analysed.

**Figure 1:**
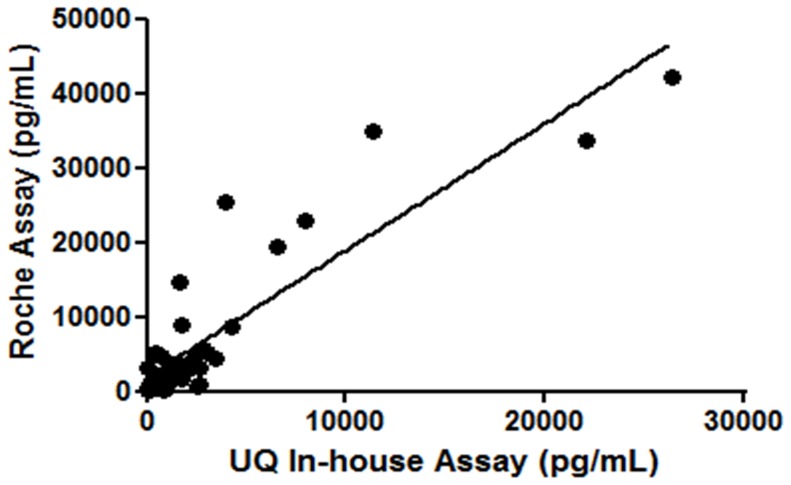
Comparison of the NT-proBNP immunoassay when compared with a commercially available diagnostic assay (Roche Diagnostics, USA). r2 = 0.78 and p<0.001.

For salivary protein analysis, unstimulated saliva is the preferred method [18], [21]. Unstimulated resting saliva was collected by the draining or drooling method described by Navazesh and Christensen [25], [26]. Volunteers were asked to rinse their mouth with water prior to donating saliva. This ensured minimal debris from food particles and that the oral cavity was well hydrated to enhance saliva production. The samples were collected and placed on ice, then transported to the laboratory on dry ice. The samples were aliquoted, de-indentified, and stored at −80°C until analysis.

**Figure 2:**
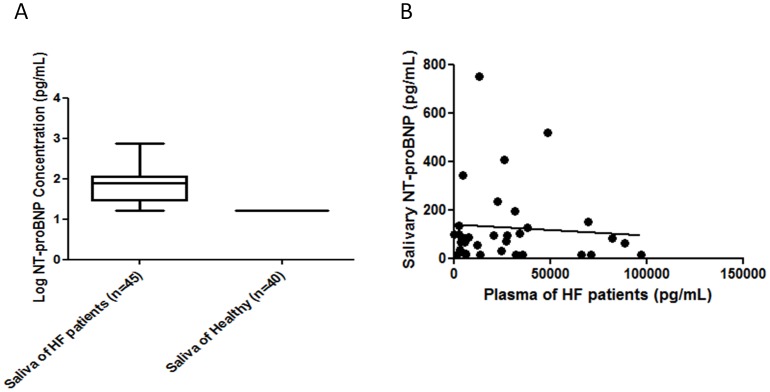
The comparison of the plasma and salivary NT-proBNP concentrations for HF patients (n = 45). (A), NT-proBNP levels in saliva of HF patients (n = 45) and healthy participants (n = 40). (B), The correlation of NT-proBNP concentrations in plasma and saliva of HF patients measured by our NT-proBNP immunoassay.

### 2.3 Salivary NT-proBNP AlphaLISA^(R)^ Immunoassay

The NT-proBNP AlphaLISA^(R)^ kit (Product-No: 1607106, *Perkin Elmer*®, *MA, USA*), was used to determine the concentrations of salivary NT-proBNP. It contains a biotinylated anti-NT-proBNP monoclonal antibody (recognising 1–12 AA sequences on the NT-proBNP analyte), which binds to the streptavidin-coated donor beads while the anti-NT-proBNP monoclonal antibody (recognising 63–76 AA) is conjugated to the acceptor beads. In the presence of NT-proBNP, the beads come into close proximity. The total reaction volume used was 10 µL. Twelve standards were used to generate a standard curve. The samples were analysed in triplicates in 384 well ProxiPlates™ (Perkin Elmer®, MA, USA). The only exception to the manufacturer recommendation was the decrease in the total reaction volumes from 50 µL to 10 µL. In summary, the immunoassay consisted of sample/analyte (1 µL), biotinylated antibody (25 mM) and acceptor bead (25 µg/mL) mix, and streptavidin donor beads (80 µg/mL). For all immunoassays, the end concentration of acceptor beads was 10 µg/mL whilst the end concentration of biotinylated antibody was 1 nM. The total incubation time was 1.5 hour at room temperature in the dark and the plates were read using an EnSpire™ plate reader (Perkin Elmer®, MA, USA).

### 2.4 The Concentration of Saliva Samples Using Amicon Filters

Saliva samples collected from both HF patients and healthy controls required concentration. Saliva (200 µL) was centrifuged using the Amicon Ultra-0.5 Centrifugal Filter Devices at 14000×g for 20 minutes. The concentrated solution was recovered by placing the Amicon filter device upside down into a clean micro centrifuge tube and NT-proBNP levels were measured using AlphaLISA^ (R)^ immunoassay.

### 2.5 Assay Performance Characteristics of the NT-proBNP AlphaLISA^(R)^ Immunoassay

#### 2.5.1 Recovery

To evaluate the suitability of AlphaLISA*^(R)^* immunoassay for measuring salivary NT-proBNP, three known concentrations of commercial recombinant NT-proBNP (Product-No: 1607106, *Perkin Elmer®, MA, USA*) were spiked in pooled saliva collected from healthy controls (n = 40). Both spiked and un-spiked pooled saliva were measured in the same AlphaLISA*^(R)^* immunoassay. The percentage recovery of the three spiked saliva sample was calculated in reference to respective un-spiked pooled saliva sample in a single AlphaLISA*^(R)^* immunoassay, using the following equation [27]:
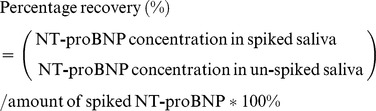



#### 2.5.2 Intra-and inter-assay coefficient of variation

To determine intra- and inter- assay variation, triplicates of saliva samples from 45 HF patients and 40 healthy controls were run in one AlphaLISA*^(R)^* immunoassay and three independent AlphaLISA*^(R)^* immunoassay, respectively [27].

#### 2.5.3 Limit of detection (LOD) of NT-proBNP immunoassay

To determine the LOD of the NT-proBNP immunoassay, 16 blanks (without the NT-proBNP analyte) were run in triplicates in one immunoassay. The LOD for the salivary NT-proBNP immunoassay was read from a sigmoidal-dose response curve based on LOD signal counts derived from the equation [28]:
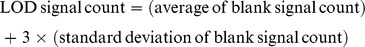



### 2.6 Statistical Analysis

All statistical analyses were performed using GraphPad Prism 5 software version 5.03 (GraphPad Software Inc., USA). A standard curve was generated by plotting the “raw” AlphaLISA counts vs the NT-proBNP standards using a 4-parameter logistic equation (sigmoidal dose-response curve with variable slope) and a 1/Y^2^ data weighting.

Kolmogorov-Smirnov statistic was performed on clinical characteristics (continuous variables) of the volunteers, in order to test for normal distribution before statistical analyses. To compare values from two groups, Mann-Whitney *U* test was performed on data without normal distribution [29] and Chi-square test for dichotomous variables. Statistical significance for differences between HF and healthy subjects was considered significant at or below p<0.05 and was calculated using the GraphPad Prism program. Pearson product moment correlation coefficients were calculated to investigate the relationship between salivary and plasma NT-proBNP levels. Diagnostic accuracy expressed as sensitivity, specificity, positive predictive value and negative predictive value were obtained from a 2×2 table with the reference standard based upon clinical diagnosis of heart failure (as described in Section 2.1).

## Results

### 3.1 Participants

In total 45 symptomatic HF patients (with left ventricular ejection fraction <40%) and 40 healthy volunteers (young, and old) were enrolled in the study. The group of HF patients consisted of 23 males and 22 females, with a median age of 73 years (ages from 53 to 88), body mass index (BMI) of 29.14, and systolic and diastolic blood pressure of 125 mm Hg and 71 mm Hg, respectively. The group of healthy controls consisted of 20 males and 20 females, with a median age of 56 years (ages from 40 to 71) and BMI of 25. Gender, age, BMI, blood pressure, heart rate, New York Heart Association (NYHA) classification of the 85 volunteers were summarised in Table 1.

### 3.2 Assay Performance for the NT-proBNP AlphaLISA^(R)^ Immunoassay

The performance characteristics of the NT-proBNP immunoassay is summarised in Table 2. Intra- and inter-assay coefficients of variation (CVs) for the NT-proBNP immunoassays were below 10%. The LOD for the salivary immunoassay was approximately 16 pg/mL.

### 3.3 Comparative Analysis of NT-proBNP Immunoassay with a Commercially Available Assay

In total, 37 plasma samples that have been previously measured for NT-proBNP levels (concentration ranges between 5 pg/mL to 42,150 pg/mL) were analysed using the two methods. The results are shown in Figure 1.

### 3.4 The Effect of Concentrating Saliva Samples

Salivary NT-proBNP concentrations from 18 HF patients were initially below the LOD of our immunoassay (16 pg/mL). Upon concentrating saliva samples, we detected NT-proBNP above the LOD (10 saliva sample from HF patients with 27.1 pg/mL to 243.8 pg/mL) and no NT-proBNP levels were detected in the filtrates. Furthermore, NT-proBNP was not detected in both concentrated saliva samples (concentrates and filtrates) collected from healthy controls who were young and old.

### 3.5 Salivary NT-proBNP Concentrations in the Healthy Control Subjects and HF Patients

The salivary NT-proBNP concentrations from the 40 healthy participants were below the LOD, <16 pg/mL. The NT-proBNP concentration in the saliva samples of the HF patients (n = 45) ranged from 18.3 pg/mL to 748.7 pg/mL with a median value of 76.8 pg/mL (interquartile range (IQR), 28.35 pg/mL to 114.7 pg/mL) (Figure 2A).

The clinical sensitivity and specificity of the salivary NT-proBNP immunoassay was 82.2% and 100% respectively, with an overall diagnostic accuracy of 90.6%. The positive predictive value for the salivary immunoassay was 100%, and negative predictive value was 83.3%.

### 3.6 Salivary vs. Plasma NT-proBNP Concentrations in the HF Population

The NT-proBNP concentration in the plasma samples ranged from 486 pg/mL to 97,319 pg/mL, with a median of 22731 pg/mL (IQR, 5386 pg/mL to 36833 pg/mL). There was no correlation between salivary NT-proBNP and plasma NT-proBNP concentrations in the HF patients (Figure 2B). The correlation of NT-proBNP concentration in plasma and saliva are as follow: n = 45; r^2^ = 0.006, p = 0.66.

## Discussion

To our knowledge this is the first time NT-proBNP has been measured in saliva samples collected from healthy subjects and HF patients. Pooled saliva from healthy control spiked with known concentrations of recombinant NT-proBNP had a recovery of 85% (Table 2). This recovery is a good indication that the NT-poBNP immunoassay is suitable for use with saliva samples. NT-proBNP was detected in the saliva samples from HF patients (sensitivity of 82.22%) but it was not detected in saliva samples from healthy control subjects. The results suggest that the presence of NT-proBNP in saliva is specific for the presence of HF. The need to concentrate 10 of the saliva samples from HF patients before the detection of NT-proBNP, suggested the presence of endogenous salivary proteins or mucins (>30 K Dalton) that could reduce the analytical sensitivity or these proteins by blocking binding sites of our bead based salivary NT-proBNP immunoassay.

Salivary NT-proBNP concentrations is approximately >200-fold lower than plasma NT-proBNP concentrations. This limitation underlines the importance of using a highly sensitive assay, such as AlphaLISA^(R)^ bead based immunoassay or possibly microchip assay systems, which enable the detection of extremely low concentrations of NT-proBNP. The poor correlation between NT-proBNP levels in plasma and saliva may suggest that the movement of heterogeneous NT-proBNP from the blood circulation into the saliva may be impaired in HF patients. Recent work by Semenov *et al.*, has indicated that HF patients tend to have a less efficient mechanism of converting proBNP (precursor molecule) by furin convertase into NT-proBNP and BNP upon secretion from cardiomyocytes into the blood circulation [30]. While furin is also present in the human saliva, its enzymatic activity in saliva is inhibited by histatins [31], which prevents in situ generation of salivary NT-proBNP. The levels of measured NT-proBNP were much lower in saliva, possibly due to the existence of a threshold level for the movement of unprocessed proBNP to saliva. Another possible explanation for the reduced sensitivity of saliva NT-proBNP to detect HF may be the presence of NT-proBNP with truncated N and/or C termini that was undetected by our immunoassay which utilised monoclonal antibodies that targeted the N (1–12AA) and C (63–76AA) termini of NT-proBNP. Kopsala *et al.*, have demonstrated that NT-proBNP in the blood circulation is extremely heterogeneous due to truncations at both termini of this molecule [8]. However, this is less likely as we observed a significant correlation between plasma NT-proBNP measured by both the Roche assay and our NT-proBNP immunoassay. Nevertheless, the result could suggest that the movement of NT-proBNP from the circulation to the saliva may vary in HF patients, and remained undetectable in the unconcentrated samples of saliva of 8 HF patients with elevated plasma NT-proBNP concentrations.

The undetected levels of salivary NT-proBNP in healthy control subjects suggest the existence of a physiological cut-off level (<16 pg/mL, the detection limit of the immunoassay) for the movement of plasma NT-proBNP into saliva. This may represent an advantage in population screening where a highly specific test is required [32]. Given that NT-proBNP is detectable in plasma samples taken from healthy control subjects [33], studies that have evaluated the utility of plasma natriuretic peptides to screen for HF precursors, such as asymptomatic left ventricular systolic dysfunction, have resulted in unnecessary, downstream testing driven by high false-positive rates [32]. Attempts to improve specificity by using urinary natriuretic peptide levels or additional biomarkers have had variable success [34], [35]. If subsequent studies confirm our findings, salivary NT-proBNP could represent a cost-effective approach to population screening by avoiding the need for phlebotomy and sample processing, and minimising unnecessary, downstream investigations.

In summary, we have demonstrated that NT-pro-BNP is detectable in saliva and that the levels were higher in a selected group of HF patients compared with healthy control subjects. Although, the correlation with plasma is not as strong, its clinical utility needs to be investigated in more detail using larger, unselected population studies before more defined cut off limits can be recommended.
